# Cholesterol and Its Derivatives: Multifaceted Players in Breast Cancer Progression

**DOI:** 10.3389/fonc.2022.906670

**Published:** 2022-05-26

**Authors:** Giorgia Centonze, Dora Natalini, Alessio Piccolantonio, Vincenzo Salemme, Alessandro Morellato, Pietro Arina, Chiara Riganti, Paola Defilippi

**Affiliations:** ^1^ Department of Molecular Biotechnology and Health Sciences, University of Torino, Torino, Italy; ^2^ Interdepartmental Center of Research in Molecular Biotechnology, University of Torino, Torino, Italy; ^3^ University College London (UCL), Bloomsbury Institute of Intensive Care Medicine, Division of Medicine, University College London, London, United Kingdom; ^4^ Department of Oncology, University of Torino, Torino, Italy

**Keywords:** breast cancer, cancer metabolism, cholesterol, mevalonate (MVA) pathway, cholesterol metabolism, statins, breast cancer therapy

## Abstract

Cholesterol is an essential lipid primarily synthesized in the liver through the mevalonate pathway. Besides being a precursor of steroid hormones, bile acid, and vitamin D, it is an essential structural component of cell membranes, is enriched in membrane lipid rafts, and plays a key role in intracellular signal transduction. The lipid homeostasis is finely regulated end appears to be impaired in several types of tumors, including breast cancer. In this review, we will analyse the multifaceted roles of cholesterol and its derivatives in breast cancer progression. As an example of the bivalent role of cholesterol in the cell membrane of cancer cells, on the one hand, it reduces membrane fluidity, which has been associated with a more aggressive tumor phenotype in terms of cell motility and migration, leading to metastasis formation. On the other hand, it makes the membrane less permeable to small water-soluble molecules that would otherwise freely cross, resulting in a loss of chemotherapeutics permeability. Regarding cholesterol derivatives, a lower vitamin D is associated with an increased risk of breast cancer, while steroid hormones, coupled with the overexpression of their receptors, play a crucial role in breast cancer progression. Despite the role of cholesterol and derivatives molecules in breast cancer development is still controversial, the use of cholesterol targeting drugs like statins and zoledronic acid appears as a challenging promising tool for breast cancer treatment.

## Introduction

Breast cancer (BC) is estimated to account for one-third of all new cancer diagnoses in American females in 2022. Despite a 1% decrease annually in mortality during the 2013-2019 timeframe, the estimated death for BC in females is 15% among all types of cancer, thus representing the second leading cause of cancer death among women (1). Molecularly, it is possible to subdivide BC into four main subtypes: Luminal BC are positive for the expression of steroid hormone receptors, the estrogen receptor (EsR) and progesterone receptor (PR), and they can be further characterized in Luminal A (EsR+, PR+, HER2-) and Luminal B (EsR+, PR+, HER2+). HER2+ BCs overexpress the HER2/ERBB2 oncogene and include both the Luminal B and the HER2+, EsR-, PR- patients. In contrast, Basal-like or Triple-Negative BC (TNBC) lacks both the hormonal receptors and the HER2 receptor (2), which represents a major obstacle for therapeutic intervention in this aggressive BC subtype.

Several epidemiological and genetic studies have tried to determine whether levels of circulating lipids are associated with risks of various cancers, including BC. Dietary cholesterol represents a significant risk factor for BC, as suggested by a comprehensive meta-analysis study (3) and genetically elevated plasma high-density lipoprotein (HDL) and low-density lipoprotein (LDL) levels appear to be associated with increased BC risk (4). However, additional studies are required to address the putative causal relationship between BC and cholesterol, with the goal to develop potential therapeutic strategies aimed at altering the cholesterol-mediated effect on BC risk.

Metabolic reprogramming has been extensively proved to be a key cancer hallmark (5); indeed, tumor cells exhibit metabolic abnormalities required to satisfy their growth and survival needs (6). Compared to more investigated metabolic phenotypes and metabolites such as glucose in the Warburg effect (7, 8), the contribution of cholesterol in cancer is still controversial (9, 10). To date, it is well known that frequently altered oncogenes and tumor suppressors in BC, like the PI3K and p53, affect cholesterol homeostasis in a variety of tumors (11–13). Interestingly, several BC samples showed increased expression of proteins involved in endogenous cholesterol synthesis, which occurs through the mevalonate (MVA) pathway (14). Moreover, BC cells display aberrant cholesterol uptake at mitochondrial levels *via* increased expression of STAR and STARD3 proteins, essential for regulating cholesterol import to the mitochondria, that, in turn, impinge on proliferation, metastasis, and survival (9, 15). Indeed, STARD3 is overexpressed in BC patients, where it is frequently co-amplified with HER2; high STARD3 levels correlate with a poor prognosis and lower response to Trastuzumab (16), a monoclonal antibody that targets the HER2 receptor (17). These data suggest a central role of mitochondria in such metabolic reprogramming.

The up regulation of cholesterol metabolism in BC cells depicts a scenario in which cholesterol and its derivatives may play a crucial role in sustaining tumor growth, hence numerous clinical trials have tried to investigate the effect of drugs able to reduce circulating cholesterol, like statins, in several cancer types. Notably, the use of cholesterol-lowering drugs in preventing or curbing BC progression has revealed controversial results (11, 18) and the ongoing clinical trials will provide a clearer view on their beneficial role. By using robust and routinely available techniques both the luminal and basal breast cancer phenotypes have shown to contain distinct subgroups and therefore to be heterogeneous (19). The single cell-based approaches to depict the BC intratumor heterogeneity, will also help in defining the co-existence of different clones in a given tumor, may help characterize distinct metabolic phenotypes and drug responses (20, 21). Nevertheless, statins treatment is a safe approach in lowering cholesterol levels (22) and the hormone dependency of BC appears to be the most promising predictive marker of response to statin treatments, probably due to the precursor role of cholesterol in steroid hormones production (11). This review will focus on the cholesterol homeostasis aberrations in BC and the relevance of MVA pathway inhibitors in BC therapy.

### Cholesterol Homeostasis

**Figure 1:**
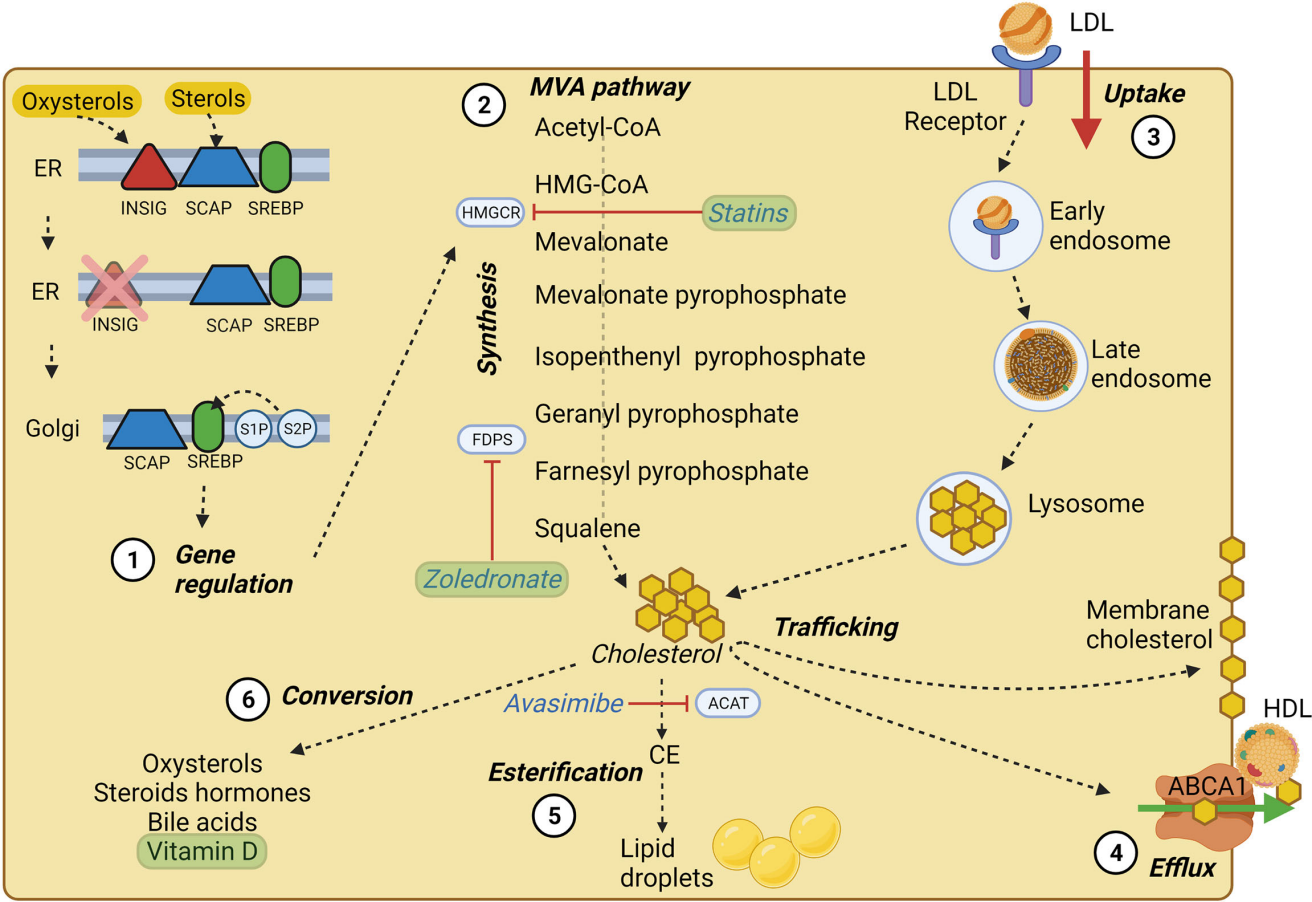
Cholesterol homeostasis main processes. (1) SREBP processing at the ER membrane and Golgi apparatus; in high cholesterol condition, SREBP is retained at the ER membrane by INSIG and SCAP, which sense oxysterols and sterols, respectively. At low cholesterol condition, SREBP can be transported to the Golgi apparatus and cleaved by S1P and S2P proteases. Cleaved SREBP can enter the nucleus and trigger the transcription of crucial MVA pathway genes. (2) Main steps of cholesterol synthesis through the MVA pathway, of which HMGCR represents the rate-limiting enzyme. (3) LDL-cholesterol intake *via* LDL-receptor mediated endocytosis. (4) Cholesterol efflux by ABCA1 transporter, which employs ATP molecules to deliver cholesterol and lipids on apoA-I, triggering the assembly of nascent HDL. (5) ACAT enzyme mediated cholesterol esterification to fatty acids tightly packaged and stored in the core of intracellular lipid droplets, which represent a ready storage of lipids that can be used without investing energy in biosynthesis. (6) Cholesterol conversion in its main derivatives, some of which may play a role in BC pathology and progression. The main cholesterol homeostasis inhibitors and their targets are underlined in blue, while drugs and substances used in clinical trials (see **Table 1**) are shown in green. Created with BioRender.com.

Cholesterol is a lipid molecule crucial for the viability of mammalian cells. It is involved in the synthesis of steroid hormones (23), bile acids (24) and oxysterols (25), and its localization in cell membranes is critical in dictating membrane integrity and fluidity (26). Cellular cholesterol results from *de novo* cholesterol synthesis and dietary intake with an estimated ratio of 70:30 (27) (**Figure 1**). The synthesis, uptake, efflux, and cholesterol conversion is tightly regulated intracellularly (28). Cholesterol is primarily synthesized in the liver and transported to other tissues through the bloodstream as an LDL-bound form. Exogenous cholesterol is mainly derived from LDL, and thanks to the LDL receptor (LDLR)-mediated endocytosis, LDLs are up-taken and stored in the early endosome (28). In the late endosome, thanks to the lipase activity, LDL undergo hydrolysis, and the derived cholesterol arrives either directly to the plasma membrane (PM) or to the endoplasmic reticulum (ER) (29), where it becomes available for esterification (30). The exit of cholesterol from late endosomes critically depends on the two cholesterol-binding proteins, NPC1 and NPC2 (29, 31, 32).

In addition to dietary intake, in nucleated cells, nearly 30 enzymatic reactions led to the polymerization of acetyl-CoA into cholesterol through the MVA pathway (18, 27). The intracellular cholesterol pool generated by the MVA pathway is controlled by two rate-limiting enzymes: 3-hydroxy-3methylglutaryl-CoA (HMG-CoA) reductase (HMGCR) and squalene epoxidase (SQLE) (27). Indeed, the homeostasis of intracellular cholesterol metabolism is mainly controlled through the transcriptional regulation of the HMGCR coding gene by the Sterol Regulatory Element-Binding Proteins (SREBPs) transcription factors, mainly by the SREBP-2 isoform in the liver. Whenever cholesterol levels at the ER membrane are high, cholesterol itself can bind the sterol sensing domain of the SCAP chaperones, while oxysterols such as 25-hydroxycholesterol can bind the INSIG chaperones at the ER membrane. INSIG and SCAP bind each other and retain SREBPs at the ER membrane (33–35). In case of low cholesterol level, INSIG is degraded, and the SCAP/SREBP2 complex can be packed into COPII-coated vesicles and targeted to the Golgi where SREBP can be proteolytically cleaved by site-1 protease and site-2 protease (S1P and S2P) (35, 36). The N-terminal domain of SREBP resulting from cleavage can enter the nucleus, bind to sterol responsive elements (SREs) and act as transcription factors, increasing the expression of LDLR, HMGCR, and SQLE, thus enhancing cholesterol synthesis and uptake (23, 36).

Cholesterol homeostasis does not rely only on its endogenous synthesis or uptake from the diet; indeed, cholesterol is heavily transported between subcellular membranes, and such trafficking may be the result of vesicular transport, membrane contact sites, or sterol transfer proteins (27). Additionally, cholesterol molecules can be esterified to fatty acid chains within the ER by the acyl-CoA cholesterol acyltransferase (ACAT) and stored into lipid droplets (37) (**Figure 1**).

### The Impact of Circulating Cholesterol in BC

The scientific community has for a long time attempted to elucidate the relationship between BC development and serum cholesterol in terms of association and causality. A plethora of investigations conducted in humans has interrogated the link between BC, LDL and HDL. Some authors have reported that high LDL levels are associated with increased BC risk (38) and are predictive of poor prognosis (39). Nevertheless, additional studies showed no association between LDL and BC risk (40–42). Concerning the prognostic value of HDL, some evidence suggests an association between low HDL and BC risk (43), especially in premenopausal women (41, 44, 45). Moreover, a retrospective study found that decreased HDL levels in pre-operative patients had a significant association with worse overall survival (46). However, others suggest that low HDL is associated with an increased risk of postmenopausal breast carcinogenesis (47).

Overall, different studies have generated contrasting results, possibly due to the multifactorial etiology of BC, its heterogeneity, and the differences in the design of the studies (48, 49). Because of the discrepancies that have emerged from clinical investigations, it is crucial to understand the potential mechanisms underlying the role of lipoproteins in BC leveraging on both animal and *in vitro* studies.

### The MVA Pathway Aberrations in BC

The MVA pathway is crucial in cell viability, not only due to cholesterol synthesis, but also because the metabolites generated through such anabolic pathway represent potential building blocks to meet the high proliferative requirements of cancer cells (11). The intracellular levels of MVA metabolites, as previously cited, are tightly controlled mainly by SREBP proteins and corresponding sterol regulatory elements (SREs). SREBPs activities can get integrated into cellular signaling pathways from growth factors and some of them are known to play a major and driver role in tumorigenesis. Among them, the PI3K-AKT signaling pathway triggered by the epidermal growth factor receptor (EGFR), is the most altered one in cancer (50). PI3K phosphorylates AKT which in turn can induce the activation of the mechanistic target of rapamycin complex 1 (mTORC1) *via* inhibition of TSC1-2 (51). Upregulation of SREBPs, caused by the PI3K/Akt signaling and mTORC1, have been associated to cancer (52) and several inhibitors of SREBPs, that are under clinical studies, proved to reduce the tumour growth in various tumor types, including BC (53).

Mutations to the catalytic α subunit of PI3K (PIK3CA) are found in 40% of Luminal A breast tumors (54). In breast epithelial cells, expression of oncogenic PI3K correlates with induced *de novo* lipogenesis *via* AKT and mTORC1 (55). Moreover, mTORC1 signaling was shown to increase RNA and protein levels of SREBP targets in primary human breast cancer samples (55). Activated AKT promotes SREBPs released from the ER by decreasing the sterol binding ability of INSIG chaperones at the ER membrane in human hepatocellular carcinoma (HCC) (56) in this model, the downstream effector of AKT, the phosphoenolpyruvate carboxykinase 1 (PCK1), once activated, can phosphorylate and promote the proteasomal degradation of INSIG, thus leading to increased SREBP maturation (56). Interestingly, PCK1 was shown to be upregulated in BC samples and to play a key role in tumor metastasis (57). Regarding mTORC1, it has been shown that its signaling may enhance SREBP maturation through the phosphorylation and activation of the downstream effector ribosomal S6 kinase 1, *via* an unknown mechanism (58). Highlighting the relevance of the mTORC1 signaling in MVA pathway aberrations and in BC, one of the downstream effectors of S6K, the ribosomal S6 is, indeed, highly phosphorylated in BC samples (55).

Interestingly, mTORC1 signaling protects BC cells from ferroptosis a cell death caused by the iron-dependent accumulation of lipid reactive oxygen species (59), by increasing SREBP1. In HER2+ cell lines bearing constant activation of PI3K-AKT-mTORC1 axis, the genetic ablation of a SREBP1 gene (SREBF1) decreased primarily the lipid synthesis-related gene SCD1, while pharmacological inhibition of SCD1, sensitized BC cells to ferroptosis (60). The antioxidant role of SCD1 is not new (61) and the mechanistic explanation may come from the role of SCD1 in producing monounsaturated fatty acid (MUFAs) (62). MUFAs can decrease lipid peroxidation sensitivity and, therefore ferroptosis, by displacing the more easily oxidized polyunsaturated fatty acids (PUFAs) from the cell membrane (63). Interestingly, SCD1 is enriched in almost all tumor tissues with a greater enrichment of SCD1 in BC compared to other tumours and to their non-neoplastic counterparts (64).

Additionally, mTORC1 may promote the chromatin accessibility of SREBPs by inhibiting Lipin 1, a phosphatidic acid phosphatase (65). Taken together, the constant activation of the PI3K-AKT-mTORC1 axis increases SREBPs translocation in the nucleus and its stabilization onto chromatin to boost the MVA pathway and increase apoptosis resistance. MYC is another well-known oncogene that is highly mutated in BC (66). MYC can interact with SREBPs and promote cell dedifferentiation (67). Notably, the SREBP2-dependent increase in cholesterol synthesis is associated with stemness maintenance and proliferation in intestinal stem cells (ISC); indeed, despite the mechanism has not been elucidated, abnormalities in phospholipid bilayer caused by the absence or inhibition of the phospholipid-remodeling enzyme LPCAT3, increases SREBP-2 nuclear activation and intestinal stem cell growth (68). Such results highlight a putative link between phospholipid content, cholesterol synthesis and stemness. As a matter of fact, stemness is a cell state that appears to be widely spread in TNBCs (69).

In 80% of TNBC cases, the tumor suppressor p53 is mutated (70). p53 null cells and mice were found capable of increasing the MVA pathway *via* inhibition of the retrograde transport of cholesterol from the PM to the endoplasmic reticulum controlled by the cholesterol transporter ABCA1 (71, 72). Mechanistically, decreased cholesterol transport from the PM to the ER results in increased maturation of SREBPs.

In BC, evidence of molecular mechanisms responsible for increased cholesterol biosynthesis is fewer than in other tumor types. The oncogenic players known to boost the MVA pathway in these tumors are also crucial in BC, where they may play similar roles. Indeed PI3K, p53 and MYC are known to modulate the MVA pathway in different tumor models and belong to the ten most frequently mutated genes in BC (73), strongly suggesting that BC cells may exploit them to upregulate cholesterol synthesis and fulfil their proliferative requirements. Interestingly, many studies identified HMGCR and SREBPs as prognostic markers in BC; in a cohort of 82 BC patients, high levels of SREBP-1 are associated with metastatic features and poor survival (74). Also, SREBP-1 knockdown negatively influences the migration and invasion of BC cells (74).

On the other hand, clinical data regarding the predictive value of HMGCR are much more controversial. Since the fact that high HMGCR expression is associated with better clinical outcomes (75, 76), is still debatable (77), further studies on larger cohorts may define a clearer scenario on the prognostic value of HMGCR.

## The Roles of Membrane Cholesterol

Cholesterol is an essential constituent of membranes, where it accounts for about 25% of total lipids (78, 79). Cholesterol plays a pivotal role in modulating PM integrity and intracellular signal transduction by interacting with specific proteins and several phospholipids and sphingolipids (80, 81). The cholesterol molecule contains a small polar hydroxyl group, a rigid steroid ring, and a flexible hydrocarbon tail. Due to its unique structure and biophysical properties, cholesterol is well-suited to pack its bulky sterol ring against the fatty acyl chains of phospholipids, leading to increased packing density and cohesion of adjacent lipids, therefore shifting from the lipid membrane liquid-crystalline state to a more ordered state (82). Alteration in the motional freedom of lipids and proteins in the PM is a major trait of cancer cells that may affect various biological processes such as the response to chemotherapeutic drugs (83–85) the activity of membrane receptors (86–88), cell motility and metastasis (89–92).

In addition to providing integrity of cell membranes, cholesterol is the major lipid component of specific membrane microdomains, named lipid rafts that range between 10 to 200 nm in size and are known to compartmentalize various cellular processes. The lipid raft concept was proposed in 1997 by Simons and Ikonen (93). It defines the lipid rafts as a dynamic clustering of sphingolipid and cholesterol within the PM that can selectively recruit and concentrate proteins while excluding others, creating a specialized membrane environment that functions as a platform for receptor trafficking and signal transduction (94). The consensus within the context of cancer cells is that lipid rafts contribute to the positive modulation of signal transmission implicated in diverse cancer cell processes, such as cell adhesion, migration, invasion, metastasis, and angiogenesis (95–97).

Increasing cholesterol levels in the PM may affect the permeability of certain metabolites and drugs, including anticancer agents (98, 99). Recently, Rivel and coworkers studied the permeation of the chemotherapeutic drug cisplatin through PM models. In this context, the increase in relative cholesterol concentration in the range of 0% to 33% induced the stiffening of lipid tails, leading to decreased drug permeability by one order of magnitude (98). Importantly, BC cells that are resistant to doxorubicin exhibit higher levels of sphingomyelin and cholesterol in the cell membrane and an increased lipid packing density than the corresponding doxorubicin-sensitive cells (100). Another study demonstrated how reducing membrane cholesterol content in BC cells could increase the efficacy of tamoxifen treatment by improving its membrane permeability (101). Therefore, the reduced drug permeability driven by increased membrane cholesterol levels may represent a strategy for cancer cells to induce drug resistance. Moreover, it is worthy of note that PM cholesterol might provoke specific conformational changes in ATP-binding cassette (ABC) transporters that are involved in multidrug efflux, potentially modulating their activity, as discussed below (83).

Researchers have paid interest in the role of cholesterol in the modulation of cancer cell migration. Overall, the general idea is that altering cholesterol abundance in cancer cells would likely affect cellular architecture and signal transduction, thus, interfering with the migratory ability of cells. It is widely recognized that lower levels of cholesterol in the plasma membrane enhance membrane fluidity and therefore favor cancer cell migration, which might eventually promote dissemination (91, 102, 103). In support of this idea, a research work from Zhao and colleagues highlighted how membrane fluidity is causally correlated with metastatic capacity *in vivo* and that many antimetastatic drugs function by inhibiting fluidity of cancer cells (103). Besides inducing membrane rigidity, cholesterol has been indirectly implicated in cell migration by affecting the stability and localization of specific proteins into lipid rafts (102, 104, 105). For instance, the presence of the transmembrane glycoprotein cluster of differentiation 44 (CD44) to lipid rafts impairs the interaction of CD44 with its migratory binding partner ezrin, leading to inhibition of BC cell migration (104, 105). In line with the anti-migration role of membrane cholesterol, another study reported that repressing cholesterol abundance in the cell membrane activates TGF-β receptor signaling, promoting metastasis of BC (102). In this work, the authors showed that mild depletion of membrane cholesterol by using low dosages (0.3 mM in MDA-MB-231 cells) of the cholesterol-depleting agent methyl-β-cyclodextrin (MβCD) led to increased cell migration and hypothesized that further cholesterol reduction might negatively influence cell survival pathways rather than promoting migratory ability of cancer cells. However, a recent study highlighted that disrupting lipid rafts in TNBC cells by using MβCD at a concentration of 0.1 mM for 48 hours is sufficient to determine up to 20% of cytotoxicity (106, 107).

On the other hand, many studies support the positive role of cholesterol-rich lipid rafts in cancer progression, since disrupting lipid rafts by using MβCD can effectively promote cancer cell death in several types of cancer cells, including BC (97, 101, 108, 109). Among the lipid-raft associated proteins whose signaling pathways contribute to more aggressive and invasive behavior of BC cells are the ion channels SK3 and Orai1 (110), the GPI-anchored cell membrane receptor uPAR, the matrix metallopeptidase protein MMP-9 (111), and the glycoprotein Muc-1 (112). Remarkably, disruption of lipid rafts by treating cells with MβCD inhibits the formation of Caveolin-1-dependent invadopodia during BC cell invasion (113, 114). In a recent study, cholesterol was found to promote the maintenance of surface levels of HER2. In this context, reducing cholesterol levels in the PM leads to the endocytic degradation of HER2, synergizing with the tyrosine kinase inhibitors to curb HER2-positive BC growth (86).

### Plasma-Membrane Cholesterol, Cholesterol Efflux and ABC Transporters

The increased amount of cholesterol incorporated in plasma-membrane also determines an increased rigidity of the membrane detergent-resistant membrane (DRM) domains and lipid rafts, which are rich in the ABC transporters -as ABCB1 (also known as P-glycoprotein, Pgp), ABCC1 (multidrug resistance-related protein 1, MRP1) and ABCG2 (BC resistance protein, BCRP), involved in the efflux of multiple chemotherapeutic drugs (115) in different tumors, including BC (116). A rigid membrane forces the transporters to assume a conformation that grants the highest catalytic capacity (83). Not only the increased endogenous synthesis (116), but also the increased uptake of LDL (8) is a typical feature of chemoresistant cells. This feature has been exploited to find an Achille’s heel to overcome drug resistance, by producing LDL-masked doxorubicin that acts as a Trojan horse to deliver the drugs within the cells (117).

The increased rigidity is not the only mechanism by which cholesterol causes chemoresistance. Indeed, oxisterols activate the transcription factors SREBP1 which cooperates with HIF-1α in up-regulating ABCB1 (118), and Liver X receptorβ (LXRβ), that increases both ABCB1 and ABCG2 at transcriptional level in ovarian cancer cells (83). Although part of conserved mechanisms, the activation of specific transcription factors is tumor specific: indeed, in TNBC and EsR-negative BC patients, LXRα, a second isoform that can be activated by oxysterols, is associated with the high expression of ABCB1 (119). Moreover, the upstream metabolites farnesyl pyrophosphate (FFP) and geranylgeranyl pyrophosphate (GGPP), synthesized in the MVA pathways, activate the signalling pathways Ras/ERK1/2/HIF-1α and RhoA/ROCK/HIF-1α, up-regulating ABCB1 and determining resistance to doxorubicin in BC (116).

Collectively, these observations sustain the direct correlation between chemoresistance and high endogenous synthesis of cholesterol, supported by the review of Tissue Cancer Genome Atlas (TCGA) (9) and on Gene Ontology (120) and Ingenuity Pathway (121)-based analysis.

Cholesterol is mainly effluxed by another ABC transporter, ABCA1, which delivers cholesterol and lipids on apoA-I and triggers the assembly of nascent HDL (**Figure 1**), followed by the efflux of cholesterol by ABCG1 and its delivery on apoE (122). Sporadic observations correlated ABCA1 to pro-tumor (103) or tumor suppressive (123) functions in cancer, and the pathways involved in ABCA1 regulation in cancer cells are still poorly explored. In dendritic cells, the high cholesterol synthesis, associated with an increased ER stress induced by cholesterol accumulation, and the inhibition of PI3K/Akt/mTOR axis, which constitutively blocks LXRα, regulate the expression of ABCA1 (124). Given the presence of aberrant activation of PI3K and Akt, caused by oncogenic mutations, we cannot exclude that this mechanism is also important in regulating ABCA1 expression and cholesterol efflux from BC cells. Together with cholesterol, ABCA1 also effluxes another isoprenoid metabolite of the MVA pathway, the isopentenyl pyrophosphate (IPP). IPP is a strong endogenous activator of Vγ9Vδ2 T-cells (125), a T-cell subset that plays a key role in anti-tumor immunity and is considered a good prognostic factor when present in the bulk of solid tumors (126). The ABCA1/apoAI system is now regarded as a useful tool to increase the activation of the host immune system Vγ9Vδ2 T-cells (127–129). Upregulating this system, by oxysterols activating LXRα, could represent a safe and effective way to boost the anti-tumor immune-response against BC tumors, where the presence of a cytotoxic T-cells infiltrate is usually associated with better prognosis and better response to the immunogenic cell death elicited by neoadjuvant or adjuvant chemotherapy (130–132).

## Cholesterol Esterification and Fatty Acids Storage in Lipid Droplets

Cholesterol esterification to fatty acids tightly packaged in the core of intracellular lipid droplets or circulating lipoproteins is a well-assessed mechanism for storage and transport of cholesterol molecules, also used to prevent cellular toxicity caused by the excess of free cholesterol (133).

Lecithin-cholesterol acyltransferase (LCAT) is a glycoprotein synthesized by the liver and secreted in the plasma. The LCAT enzyme is responsible for the synthesis of cholesterol esters in plasma and, together with cholesteryl ester transfer protein (CETP), plays a critical role in the maturation of high-density lipoproteins (HDL), helping to determine their composition, structure, metabolism and plasma concentration (134).

At an intracellular level, cholesterol esterification is accomplished by two sterol O-acyltransferase enzymes: Acetyl-CoA Acetyltransferase 1 (ACAT1), which is widely distributed in all tissues, and ACAT2, which is preferentially expressed in the liver and the intestine. Both enzymes play a key role in cellular cholesterol homeostasis, using long-chain fatty acyl-coenzyme A as the fatty acyl donor to convert cholesterol to cholesteryl esters (CE) in the cytoplasm, leading to lipid droplets formation. Their main function is to avoid cell toxicity due to an excessive accumulation of free cholesterol in cell membranes (135). However, ACAT is highly expressed in some tumors, and its expression is reported to be activated by several factors such as IFN-γ, TNF, and insulin, but not by cholesterol and fatty acids, which are indeed able to mediate ACAT2 proteasomal degradation through reactive oxygen species (ROS) induction (18).

Lipid metabolism gene expression resulted to be also impaired in BC cells in comparison to the regular surrounding tissues (136, 137). In fact, a high ACAT expression leads to a faster recovery of BC cells proliferation upon nutrients deprivation. TNBC cells have been observed to have an enhanced CE synthesis and storage. The inhibition of ACAT1 reduces LDL-induced both proliferation and migration in these cells (138, 139).

Proliferating BC cells, in fact, need a constant lipid supply, which can derive both from a *de novo* synthesis or exogenous cholesterol and fatty acid uptake from plasmatic LDL, leading to increased storage in cytoplasmic lipid droplets (139). The lipid-accumulation represents a lower energy-consuming strategy, as lipid droplets represent a ready storage of lipids which can be used without investing energy in biosynthesis.

Some *in vivo* studies showed a correlation between intratumor CE accumulation and Ki-67, a well-known marker of tumor cell proliferation, poor patient survival, and higher risk of relapse. Furthermore, there is some evidence of a causal relationship between CE and BC. Exogenous and endogenous CE can increase mammary tumor growth and ACAT1 may be a potential target for the treatment of BC (140).

CE accumulated in lipid droplets have been correlated also with resistance to chemotherapy. After an acute exposure to doxorubicin, chemoresistant clones of TNBC increased both mitochondria, induced by the peroxisome proliferator activated receptor α (PPARA) and γ (PPARG) proteins, and lipid droplets. Overall, these changes shift the metabolism toward oxidative phosphorylation (OXPHOS), supported by the accumulation of fatty acids in the CE of LD (141) and antagonized by perilipin 4 (PLIN4). Interestingly, high intratumor levels of PPARA, PPARG and PLIN4, and consequently of CE and lipid droplets, are new biomarkers predicting resistance to neoadjuvant chemotherapy in TNBC (141).

## Cholesterol Derivatives and Their Roles in BC: (Hydr)oxy Sterols, Steroid Hormones and Vitamin D

Not only cholesterol, but also its derivatives, may play a role in BC pathology and progression. Here, among all, we report the following:

### (Hydr)oxy Sterols

Oxysterols are cholesterol metabolites that can be synthesized through oxidation by both enzymatic reactions and radical processes. They are involved in several cellular functions and physiological processes, such as the modulation of membrane fluidity and cholesterol metabolism and transport, but also in BC pathology and progression (142). Moreover, oxysterols have been described to be LXR-specific ligands. Some oxysterols are implicated in tumor formation (143), as recent data put in correlation their plasma levels in BC patients with clinical data (144), while others are considered anti-tumor agents (49).

For instance, 25-hydroxycholesterol (25-HC) and 27-hydroxycholesterol (27-HC) have been shown to enhance EsR expression in estrogen-deprived BC cell lines, suggesting that oxysterol are able to substitute estrogen in receptors activation and can play a potential role in resistance to the therapy (142, 145). In fact, 25-HC and 27-HC have been associated with resistance to aromatase inhibitors, which block estrogen synthesis but do not affect EsR expression. Indeed, BC patients treated with aromatase inhibitors had significantly increased plasma levels of 27-HC and (even if more moderately) 25-HC after treatment (146), supporting the potential role of 25-HC and 27-HC level and therapy outcome of patients (142). Accordingly, 25-HC, has been found elevated in the circulation of BC patients who have relapsed compared to those with primary disease (146).

In particular, 27-HC is produced by the cytochrome P450 27A1 (CYP27A1) enzyme, of particular interest in BC. It is, in fact, highly expressed among patients with high tumor grade, i.e., with less differentiated tumor cells (142, 147), and some *in vivo* experiments indicate that it could be a potential target for BC treatment (148). Accordingly, it has been reported that high levels of CYP7B1, a cytochrome p450 enzyme responsible for the catabolism of 27-HC, are associated with better survival outcomes in mice (49, 147).

Moreover, upon 27-HC exposition, BC cells showed increased proliferation and growth (18, 142). Additionally, 27-HC promotes BC cells migration and metastasis by affecting tumor microenvironment (18) through the recruitment of immune suppressive neutrophils in the metastatic niche (149). Consistently with these data, Moresco and colleagues demonstrated that oxysterols depletion reprograms the tumor microenvironment favoring the control of breast tumors and metastasis formation (150).

Overall, these data suggest that oxysterols could be potential targets for BC therapy.

### Steroid Hormones

Cholesterol is also an important precursor of steroid hormones, many of which have clinical relevance (151). Steroid hormones are the products of steroidogenesis, a process that takes place in the mitochondria and smooth ER starting from cholesterol, which is mainly taken from LDL (152). Cholesterol is metabolized down a number of enzymatic pathways and converted to the 21-, 19-, and 18-carbon steroid hormones. Steroidogenesis starts with the transport of cholesterol into the mitochondria. This passage is controlled by the steroidogenic acute regulatory protein (StAR) (153). Subsequently, cholesterol is converted by the mitochondrial side-chain cleavage enzyme complex into pregnenolone. Pregnenolone, from which the other entire steroid hormones derive, is metabolized by several enzymes, leading to progesterone or androstenedione formation by 17-hydroxylase/17, 20-lyase enzyme. Androstenedione is further transformed into other androgens or estrogens (152).

Steroid hormones can be grouped into five categories: glucocorticoids, mineralocorticoids, androgens, estrogens and progestogens. Due to their lipophilic nature, steroid hormones cannot be stored in intracellular vesicles. As a consequence of their easy diffusion, they are synthesized as precursors and rapidly converted into active hormones when needed upon stimulation of the parent cell (151).

Of particular interest in the BC context are the ovarian hormones progesterone and estrogen, which are involved in tumor aetiology, progression and treatment. It is well assessed that a large percentage of BC are hormone-dependent, where cancer cells take advantage of local or systemic estrogens for sustaining their growth (154). In recent studies, androgens (in particular 11-oxygenated androgens) and glucocorticoids have been identified as biomarkers of BC risk, especially in women with a family history of BC, despite being much less studied (155).

The signalling events downstream hormone receptors include the direct or indirect modulation of gene expression, post-transcriptional regulation by miRNAs and signal transduction factors. Moreover, it has been described that these players act on BC stem cells (154, 156).

Furthermore, it is reported that prolonged exposure to ovarian hormones and progestin correlates with a BC risk, while progesterone and EsR are targets for advanced tumor therapy (154, 157). In fact, hormonal therapy is mandatory for all patients with hormone receptor-positive BC (158). This therapy aims to prevent estrogens stimulation of signalling pathways in cancer cells and can be performed through different strategies, including estrogens biosynthesis blockage or estrogens action through the use of agonist, antagonist or both (158).

Moreover, a close relationship between estrogen/testosterone metabolism and the MVA pathway in BC has been demonstrated. In particular, recent studies have shown that 17β estradiol and testosterone play key roles in rising MVA pathway enzymes, impacting on RAS proteins prenylation and farnesylation in several tumors, including breast and prostate cancer (159).

Taken together, this common evidence indicates that steroid hormones play an essential role in the development and classification of BC since they are commonly associated with risk and aetiology. In addition, they are potential targets for diagnostic tools (160) and BC treatment (152).

### Vitamin D

Another interesting cholesterol derivative with hormonal activity, is vitamin D. Vitamin D3 is a fat-soluble vitamin whose biosynthesis takes place in skin cells and involves the irradiation of 7-dehydrocholesterol (a cholesterol precursor in the MVA) by ultraviolet (UV) radiation. It is influenced by several factors such as the availability of 7-dehydrocholesterol and atmosphere condition, skin pigmentation and age (161).The newly synthesized vitamin D3 is further hydroxylated in the liver, by the enzyme 25-hydroxylase, to 25-hydroxyvitamin D or calcitriol, the active hormonal form of vitamin D. Once released in the extracellular space, Vitamin D3 binds to the vitamin-D binding protein, which shuttles Vitamin D through the bloodstream, and finally interacts with its receptor (VDR), which is ubiquitously expressed (162). Vitamin D can also derive from the diet, and it is an essential player in many physiological processes, including bone metabolism, cell growth and calcium and phosphorus absorption. On the other hand, pleiotropic effects of vitamin D such as anti-inflammatory and anti-neoplastic properties, are still under study (163). In particular, preclinical studies underlined that the vitamin D system has onco-protective functions, hindering several cellular processes such as differentiation, regulation of inflammation, apoptosis, proliferation, invasion and angiogenesis and metastasis formation (164).

As a matter of fact, vitamin D deficiency is one of the most common health problems worldwide (165, 166) and is a risk factor for several diseases, including metabolic syndrome (167), cardiovascular disease and cancer (162, 168). Interestingly, the first link between vitamin D and cholesterol has been described by Li et al., who demonstrated that vitamin D deficiency could enhance the amount of serum cholesterol by lowering the vitamin D receptor activity, leading to an increased cholesterol biosynthesis in the liver (168). These data appear to be consistent with another study performed by Jiang and colleagues that reported a link between vitamin D deficiency and dyslipidaemia. In particular, they described an inverse correlation between vitamin D and LDL cholesterol/triglycerides levels, while they demonstrated a positive association with the HDL cholesterol level (169).

The relationship between vitamin D and BC has been extensively studied and its role in tumor progression is well assessed (170, 171). In particular, it has been described an association between the impaired vitamin D and VDR molecular pathway and tumorigenesis in breast tissues (172), while VDR levels inversely correlates with a most aggressive tumor phenotype. Hence, VDR is considered a favourable prognostic factor and associated with a lower risk of BC death, supporting the protective anticancer role of vitamin D (164, 173, 174). Consequently, preclinical, clinical and epidemiological studies have established that vitamin D deficiency is a risk factor for BC development (175, 176).

Interestingly, calcitriol exhibited antiproliferative effects in BC cell cultures and delayed tumor growth in animal models of BC through different mechanisms (177). In particular, due to its anti-inflammatory activity and ability to suppress estrogen biosynthesis by down-regulating ERα expression, its potential therapeutic utility has been suggested in combination with other drugs in EsR+ BC patients (177).

Furthermore, recent studies speculate about vitamin D inducing molecular mechanisms able to reverse drug resistance in several tumors, including BC. Thus, many authors suggest using calcitriol in combination with anti-cancer drugs to potentiate BC therapy (178, 179).

Numerous randomised clinical trials attempted to define the efficacy of vitamin D supplementation in BC outcomes (Tab. 1). However, despite the promising results from observational studies, none of these trials could confirm reduced cancer-related mortality among cancer patients (180).

## Classic MVA Pathway Inhibitors and BC Therapy

### Statins

Dysregulation of the MVA pathway is a relevant lipid reprogramming often observed in BC. Several trials and epidemiologic studies support an inverse correlation between the use of MVA inhibitors, such as statins, and mortality rate in BC (181). Statin class of drugs has been largely used to lower blood cholesterol levels, by inhibiting the core HMGCR enzyme of the MVA pathway, in particular for cardiovascular diseases treatments. During the last years, several epidemiologic and clinical research studies underlined their beneficial role in concomitant diseases such as BC, even though an exact mechanism in this context is not yet fully understood (182, 183).

Despite some studies suggesting no close association between statin use and BC risk (184, 185), recent evidence showed a link between statin use and reduced recurrence and disease-specific mortality in BC patients, with an improved BC prognosis and survival (186–188).

Interestingly, Beckwitt and colleagues in their work demonstrated that statins are able to interfere with metastatic cascade and suppress metastatic BC outgrowth, suggesting that this class of drugs could be a potential long term adjuvant in order to prevent dormant BC micro-metastasis, which are responsible for the majority of BC deaths (189).

Moreover, a positive correlation between statins treatment and some clinical benefits in TNBC was observed in women starting statins therapy within one year after the diagnosis (190).

In particular, recent preclinical data describe an impact for Atorvastatin in favouring chemotherapy effects in TNBC, suggesting its possible use in conjunction with metastatic chemotherapy to reduce TNBC cancer progression (191).

Taken together, these data indicate a general protective role for statins in the treatment of BC in combination with standard therapy, although completed clinical trials have provided controversial results (**Table 1**). Ongoing and future interventional studies will give a better understanding concerning the safety and the efficacy of these compounds.

**Table 1:** A list of completed interventional studies with published results that assess the beneficial role of cholesterol-lowering drugs and vitamin D in BC patients.

Target	Drug	Objectives	Results	Phase	NCT Number and References
HMGCR	Simvastatin	Identification of biomarkers modulated by simvastatin in women at increased risk of a new BC	Reduction of circulating estrone sulfate No changes in mammographic density (MD)	II	NCT00334542 (205);
		Investigating concurrent anastrozole and simvastatin treatment in post-menopausal women	Simvastatin does not compromise the activity of anastrozole	II	NCT00354640 (206);
	Lovastatin	Lovastatin effect on women with a high inherited BC risk	No significant biomarkers modulation	II	NCT00285857 (207);
	Fluvastatin	Evaluating biomarkers changes	Decreased proliferation and increased apoptosis markers	II	NCT00416403 (208);
Farnesyl Diphosphate Synthase	Zoledronic Acid	Investigating the effects on bone marrow micrometastases	Reduced abundance of disseminated tumor cells	II	NCT00295867 (209);
		Effect of ZA in combination with Letrozole in post-menopausal BC patients	Improved disease-free survival Preserved bone mineral density	III	NCT00171340 (210);
		Investigating the effect of ZA in combination with chemotherapy and/or hormone therapy	Adjuvant ZA reduced the risk of fractures	III	NCT00072020 (211);
			Improved disease-free survival in pre-menopausal patients with early-stage BC taking anastrozole or tamoxifen	III	NCT00295646 (212);
		Assess the efficacy and safety	Therapeutic effect maintained at reduced dosing frequency	III	NCT00375427 (213);
			No significant differences in disease-free survival or overall survivor Improved the bone mineral density	II	NCT00213980 (214);
		Assess the efficacy and safety in combination with Dasatinib	Combination well tolerated Indication of clinical benefit for HR-positive patients	II	NCT00566618 (214);
Vitamin D Receptor	Vitamin D	Evaluate changes in BC biomarkers	No significant changes in MD	III	NCT01224678 (215);
				I/II	NCT00976339; NCT00859651 (216);

### Zoledronate

Another MVA pathway inhibitor is zoledronate (or zoledronic acid - ZA). It is a potent and long-acting bisphosphonate drug in clinical use. It acts by blocking the farnesyl pyrophosphate synthase (FPPS) in the MVA pathway, thereby inhibiting the synthesis of cholesterol and isoprenoid lipids required for prenylation of signalling proteins (192). Clinical practice guidelines recommend the use of ZA for the treatment of early BC in post-menopausal women (193, 194), since it improves osteoclast bone resorption for the treatment of hypercalcemia of malignancies and management of bone metastasis (195) (**Table 1**). Interestingly, its potential effects in reducing cancer, cardiovascular diseases and mortality could be more important than its skeletal actions (196, 197).

However, recent evidence has shown that ZA is able to modulate signaling pathways involved in apoptosis and that could be beneficial to be used together with letrozole to treat EsR-positive BC patients (198).

Interestingly, ZA involvement in immunomodulation of tumor microenvironment has also been described. In fact, Ubellacker et al. demonstrated that a single relevant dose of ZA is able to generate BC suppressive bone marrow cells, which could concur in a reduction of breast tumor development and progression (199). Moreover, ZA seems to explicate an anti-tumor activity enhancing the proliferation, migration, and immunosuppressive function of T-regulatory cells (Tregs) by affecting Tregs interaction with BC cells and synergistically acting with cytokine or IDO inhibitors leading to enhanced anti-tumor immunity (200).

Another benefit of ZA treatment is overcoming BC cells chemo-resistance due to the induction and activation of apoptosis pathway. In fact, BC stem cells, considered mainly responsible for tumor recurrence and drug resistance, decrease their viability in a dose- time-dependent manner upon ZA exposition (201). In correlation with these data, Jia and colleagues described ZA inhibition on ERK/HIF pathway leading to a higher drug sensitization in EsR-positive BC (202).

However, some data demonstrated that ZA does not increase disease-free survival, despite improving the pathologic complete response, thus might not being sufficient to ameliorate post-menopausal patient outcomes in HER2-negative BC (203).

Nevertheless, ZA is the object of clinical studies with other types of bisphosphonates (204).

Taken together, the available data indicate a general protective effect of MVA pathway inhibition with drugs in BC (**Table 1**). Despite any case and effect needing to be individually evaluated, it could be an interesting adjuvant tool in BC therapy.

## Conclusion and Outlook

From the above data, a complex picture on the role of cholesterol and its derivatives in BC is emerging. The enzymes that control the various steps leading to cholesterol or derivatives synthesis and the protein involved in trafficking towards the membrane or in the uptake from the circulation are all involved in cholesterol homeostasis and can be affected by cell transformation. At the same time, they look promising as targets for antitumor drugs.

As stated in **Table 1**, current clinical trials indicate that the MVA pathway inhibition with specific drugs like statins and ZA, is protective in BC. In addition to statins and ZA, some new cholesterol metabolic molecules have recently emerged as promising drug targets for cancer treatment (18). An example comes from targeting the cholesterol esters through inhibition of ACAT1 with the potent inhibitor avasimibe. In melanoma, in the immune response to cancer, avasimibe promotes TCR aggregation and immune synapse formation in CD8+ T cells by elevating the cholesterol content of the PM, thus enhancing the killing effect of CD8+ T cells (18). Avasimibe has been proven to have a good human safety profile in previous clinical trials in the treatment of atherosclerosis (127). Therefore, targeting ACAT1 by avasimibe may be a safe and effective method to disrupt cholesterol metabolic homeostasis in cancer treatment, as it has begun to be explored in recent preclinical BC studies (217) and it would be interesting to evaluate its effects in BC clinical practice.

It is also very important to underline those synergistic effects of low doses of cholesterol inhibitors, statins or ZA, together with low doses of chemotherapy drugs, might reach the target of increased efficacy and decreased adverse effects and resistance. Therefore, at least preclinical experiments are required to set the optimal range of treatments in BC mouse syngeneic models, in which both the tumor and the tumor microenvironment with the complex immune repertoire can be explored.

BC heterogeneity and the complex cellular architecture plays a key role in drug responsiveness and resistance to therapy that are the major challenges in BC treatment of aggressive tumors, like the TNBC, and are responsible for tumor relapse. However, deciphering the neoplastic subtypes and their spatial organization is still challenging. Nowadays in addition to panels of protein biomarkers useful for classifying clinical phenotypes of breast cancer (19), the progress in single-nucleus RNA sequencing will allow the identification of cell populations and of their spatial distribution in breast cancer tissues with costs that will become more and more accessible. This could be performed in parallel with metabolomics analysis of cell populations. Data coming from these experiments will allow tracing the clonal evolution of cells that are more addicted to the MVA pathways in the tumor. Finally, coupling innovative combinatorial therapies, chemotherapy and inhibitors of the cholesterol pathways, with the analysis at a single cell level will highlight in a given BC specific different clones, which may contribute to metabolic phenotype and drug response.

## Data Availability Statement

The original contributions presented in the study are included in the article/supplementary material. Further inquiries can be directed to the corresponding author.

## Author Contributions

GC, DN, AP, and CR searched for current literature on the topic and wrote the manuscript. GC and AP designed the figure. CR and PD reviewed the manuscript and finalized it for publication. All authors contributed to the article and approved the submitted version.

## Funding

This work was supported by AIRC (Associazione Italiana Ricerca Cancro) to PD (IG-20107) and CR (IG21408), Compagnia San Paolo, Torino, Progetto DEFLECT to PD, Fondazione CRT 2020.1798 to PD.

## Conflict of Interest

The authors declare that the research was conducted in the absence of any commercial or financial relationships that could be construed as a potential conflict of interest.

The reviewer LL declared a shared affiliation with the authors GC, DN, AP, VS, AM, CR, and PD to the handling editor at the time of review.

## Publisher’s Note

All claims expressed in this article are solely those of the authors and do not necessarily represent those of their affiliated organizations, or those of the publisher, the editors and the reviewers. Any product that may be evaluated in this article, or claim that may be made by its manufacturer, is not guaranteed or endorsed by the publisher.
